# Associations between Macronutrient Intake and Obstructive Sleep Apnoea as Well as Self-Reported Sleep Symptoms: Results from a Cohort of Community Dwelling Australian Men

**DOI:** 10.3390/nu8040207

**Published:** 2016-04-08

**Authors:** Yingting Cao, Gary Wittert, Anne W. Taylor, Robert Adams, Zumin Shi

**Affiliations:** 1 Population Research and Outcome Studies, the University of Adelaide, SAHMRI, Adelaide, SA 5005, Australia; anne.taylor@adelaide.edu.au (A.W.T.); zumin.shi@adelaide.edu.au (Z.S.); 2 Freemasons Foundation Centre for Men’s Health, the University of Adelaide, Adelaide, SA 5005, Australia; gary.wittert@adelaide.edu.au; 3 Health Observatory, Discipline of Medicine, the Queen Elizabeth Hospital Campus, the University of Adelaide, Adelaide, SA 5011, Australia; robert.adams@adelaide.edu.au

**Keywords:** macronutrient intake, fat intake, apnoea hypopnea index, polysomnography, daytime sleepiness

## Abstract

*Background:* macronutrient intake has been found to affect sleep parameters including obstructive sleep apnoea (OSA) in experimental studies, but there is uncertainty at the population level in adults. *Methods:* cross-sectional analysis was conducted of participants in the Men Androgen Inflammation Lifestyle Environment and Stress cohort (*n* = 784, age 35–80 years). Dietary intake was measured by a validated food frequency questionnaire. Self-reported poor sleep quality and daytime sleepiness were measured by questionnaires. Overnight in-home polysomnography (PSG) was conducted among participants with without previously diagnosed OSA. *Results:* after adjusting for demographic, lifestyle factors, and chronic diseases, the highest quartile of fat intake was positively associated with excessive daytime sleepiness (relative risk ratio (RRR) = 1.78, 95% CI 1.10, 2.89) and apnoea-hypopnoea index (AHI) ≥20, (RRR = 2.98, 95% CI 1.20–7.38). Body mass index mediated the association between fat intake and AHI (30%), but not daytime sleepiness. There were no associations between other intake of macronutrient and sleep outcomes. *Conclusion:* high fat is associated with daytime sleepiness and AHI. Sleep outcomes are generally not assessed in studies investigating the effects of varying macronutrient diets on weight loss. The current result highlights the potential public health significance of doing so.

## 1. Introduction

A body of evidence has shown the associations between macronutrient intake and sleep parameters, however, with inconsistency. Carbohydrate, particularly with high glycaemic index (GI) was associated with faster sleep onset in healthy young men [1] but was associated with increased total arousal in children compared with low GI [2]. Low intake of protein (<16% *vs.* ≥16%) has been shown to be associated with difficulty in initiating sleep, but high intake of protein (≥19% *vs.* <19%) has been shown to be associated with difficulty maintaining sleep in middle-aged Japanese workers [3]. A fatty meal was found to aggravate apnoea in patients (overweight or obese) with obstructive sleep apnoea (OSA) [4]. A newly published randomized-crossover study by St-Onge’s group found that low fibre and high saturated fat and sugar intake was associated with lighter sleep with more arousals in young and middle-aged healthy adults [5]. However, other studies suggested no association between fat intake and sleep quality [6] or insomnia symptoms [3]. Although the inconsistent results may be attributed to a variety of study designs, uncertainty remains regarding the association between macronutrient intake and sleep in the current literature.

Studies that investigate the associations between macronutrient intakes and sleep parameters (objective measurements) at the population level in the community setting are desired. One study in Caucasian and Hispanic adolescents (*n* = 319) found that total fat intake was negatively associated in girls but positively associated in boys with rapid eye movement sleep [7]. However, there are no similar studies in adults. In this study, we aimed to assess whether macronutrient intake was associated with Apnoea-hypopnea Index (AHI) and self-reported sleep symptoms in community-dwelling middle-aged men at the population level under non-experimental conditions. 

## 2. Methods

### 2.1. Study Population

The Men Androgen Inflammation Lifestyle Environment and Stress (MAILES) cohort study was established in 2009, to investigate cardio metabolic disease risk factors in relation to sex steroids, inflammation, environmental and psychosocial factors in men. A detailed cohort profile has been published previously [8]. Briefly, the study population consists of 2563 community dwelling men aged 35–80 years at baseline (MAILES stage 1) from the harmonisation of two population cohort studies: all participants from the Florey Adelaide Male Ageing Study (FAMAS) (2002–2005) [9] and eligible male participants from the North West Adelaide Health Study (NWHAS) (2004–2006) [10]. The MAILES stage 2 (2007–2010) was an approximate five-year follow-up consisting of a Computer Assisted Telephone Interview (CATI), questionnaires and biomedical examinations. In total, 1815 men completed the dietary intake during stage 2. 

**Figure 1:**
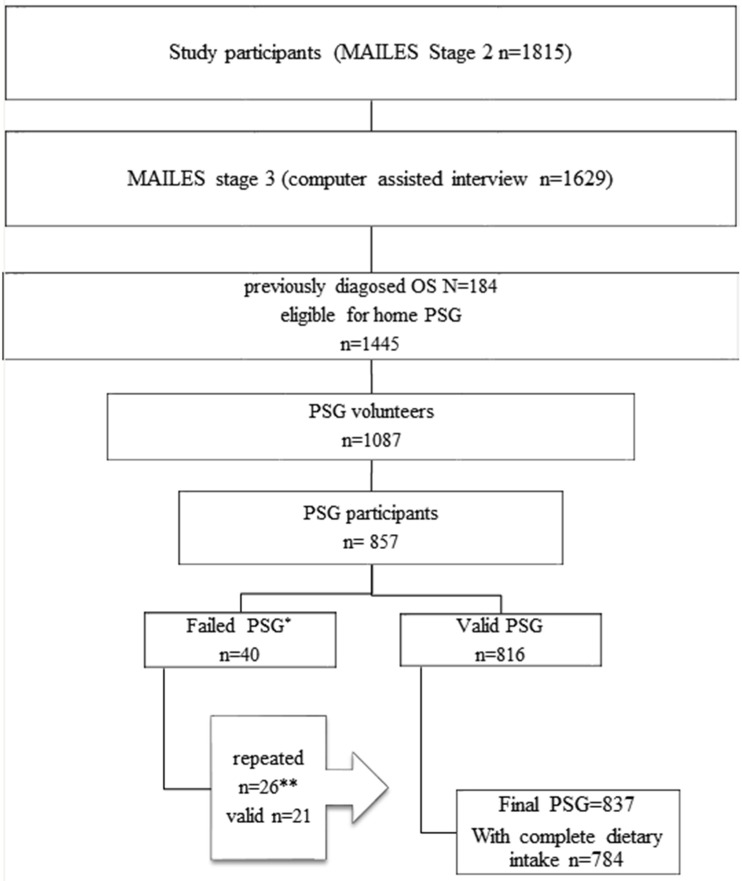
The flow chart of study participants with dietary intake (MAILES stage 2) and MAILES stage 3 with PSG recruitment * *n* = 21 total sleep time (TST) not ≥3.5 h from ≥5 h recording; *n* = 3 poor respiratory signals; *n* = 2 poor EEG; *n* = 14 no oxygen saturation (SaO_2_); *n* = 3 all traces/recording failed. ** Includes 20 successful and 3 failed second PSG of which one was successfully reperated at a third time (this flow chart with instructions for PSG recruitment has been published previously [11]).

MAILES stage 3, conducted in August 2010, consisted of a CATI including sleep related questions (*n* = 1629). The 184 who answered “yes” to the question “Have you ever been diagnosed with OSA with a sleep study” were excluded from participating in the sleep sub-study, and the 1445 men who answered “no” to the question were further asked if they were willing to participate in the sleep study (75.2% agreed). Of these, a random sample of 1087 was chosen for inclusion. A total of 857 had home based PSG (Figure 1 [11]), and 837 of them had final valid measurements and became the study population in this paper aimed at examining the association between macronutrient and AHI. Self-selection bias was examined by comparing those who underwent a sleep study with those men in the MAILES cohort who did not. Sleep study participants did not differ from non-participants in daytime sleepiness, waking frequency and obesity level but they were younger, and more likely to report frequent snoring and better general health [11]. Ethics approval was obtained from the Queens Elizabeth Hospital Human Ethics Committee for the NWHAS study (number 2010054) and the Royal Adelaide Hospital Human Research Ethics Committee for the FAMAS study (number 020305h).

### 2.2. Macronutrient Intake Assessment

Dietary intake was measured by the Cancer Council Victoria Diet Questionnaire for Epidemiological Studies (DQES-V3.1 (FFQ)). The FFQ has been validated in an Australian population and is widely used in epidemiological studies [12]. The questionnaire asks the participant’s habitual consumption of 167 foods and six alcohol beverages over the previous 12-month on a 10-point frequency scale. Additional questions were asked about the type of breads, dairy products and fat spreads used. Macronutrient intakes were computed from the dietary data by the means of the nutrient composition tables in the NUTTAB95 database (Food Standards Australia New Zealand, Canberra, Australia, 1995). 

### 2.3. Sleep Assessments

Sleep measurements consisted of subjective (CATI and self-reported questionnaires) and objective (in-home PSG) approaches. Self-reported data included: (1) the STOP (snore, tiredness during daytime, observed apnoea and high blood pressure) questions [13]; (2) the Pittsburgh Sleep Quality Index (total score ranged from 0 to 21, a score >5 indicates poor sleep quality) [14]; and (3) sleepiness asked by the question “Do you feel sleepy when sitting quietly during the day or early evening? (1) yes (2) no (3) sometimes”.

AHI was measured by a single overnight in-home PSG with Emblettas X100 portable sleep device [15]) and manually scored by an experienced sleep technician according to the 2007 American Academy of Sleep Medicine criteria (alternative) [16]. 

### 2.4. Other Measurements 

Information on education, marital status, income, work status, physical activity, smoking, shift-work, and chronic diseases were collected by questionnaires [8]. Medication use was obtained from Medicare Australia by confidential unit record linkage, classified according to the Anatomical Therapeutic Chemical (ATC) Classification. The number of distinct medication classes (at the ATC third level) six months before clinical examination were treated as covariates. 

Body weight was measured in light indoor clothing without shoes to the nearest 100 g. Height was measured without shoes to the nearest mm using a stadiometer. Waist circumference was measured to the nearest mm, midway between the inferior margin of the last rib and the crest of the ilium, in the mid-axillary line in a horizontal plane. Blood pressure was measured twice using a mercury sphygmomanometer on the right upper arm of the subject, who was seated for five minutes before the measurement.

### 2.5. Statistical Analysis 

Macronutrient (carbohydrate, protein and fat) intakes (g) were recoded into quartiles (Q1–Q4). Chi square test was used to compare difference between categorical variables, and ANOVA was used to compare differences in continuous variables between groups. The association between quartiles of macronutrient intake and self-reported sleep (snoring and poor sleep quality) was assessed using Poisson regression. Multinomial logistic regression analysis was used to test the association between macronutrient intake and self-reported sleepiness (“yes”, “sometimes” and “no”), as well as the association between macronutrient intake and AHI. AHI was divided into three categories: low (<5), medium (5–19) and high (≥20). Using low level and the lowest quartile (Q1) of each macronutrient intake as the reference group, multivariate-adjusted associations were performed: (1) model 1 adjusted for age; (2) model 2 further adjustments for education, smoking, alcohol intake, physical activity and shift-work; (3) model 3 further adjustments for waist circumference, diabetes, depression and medication. We did a sensitivity analysis by further adjusting for energy intake in model 4. Structural equation modelling (SEM) was used to test whether body mass index (BMI) mediates the association between macronutrient intake and AHI (treated as continuous variable) and daytime sleepiness (“yes” was assigned with value 2, “no” was assigned with value 0, “sometimes” was assigned with value 1, and treated as continuous variables). Direct and indirect effects were estimated using command “estimate teffects”. Linear trend across quartiles of each macronutrient intake was tested using the median value of each macronutrient intake (g) at each quartile and treating it as a continuous variable in the model. All statistical procedures were performed using STATA 13.0 (Stata Corporation, College Station, TX, USA).

## 3. Results

Overall, 1815 participants with dietary intake were analysed, of whom 837 without a prior diagnosis of OSA underwent successful sleep studies and 784 completed the dietary intake. Demographic characteristics by quartiles of each macronutrient intake of the participants are presented in Table 1. The mean age of the participants was 59.7 (SD 11.4) years. Characteristics of PSG participants with dietary intake are presented in Table S1.

Univariate analysis results between macronutrient intake and AHI and self-reported sleep parameters are presented in Table 2. No association was found between carbohydrate or protein intake and AHI. High intake of fat was positively associated with high AHI and self-reported daytime sleepiness. The prevalence of sleepiness was 46.4% and 37.0% among those with highest and lowest quartiles of fat intake. The distribution of AHI was significantly different across quartiles of fat intake with high fat intake associated with high AHI.

The prevalence ratio of self-reported sleep parameters (relative risk ratio for sleepiness) across quartiles of macronutrient intake is presented in Table 3 and Figure S1. After adjusting for age, waist circumference, education, lifestyle factors (smoking, alcohol intake, physical activity and shift work), chronic diseases and medication, the highest quartile of fat intake was positively associated with daytime sleepiness. Compared with the lowest quartile, the highest quartile of fat intake had a relative risk ratio (RRR) of 1.78 (95% CI 1.10–2.89) for daytime sleepiness (*p* for trend across quartiles was 0.305). When further adjusted for total energy intake, the association was no longer significant. There were no associations between macronutrient intakes and other self-reported sleep parameters. The RRR for AHI using multinominal logistic regression are presented in Table 4 and Figure S2. After adjusting for age, waist circumference, lifestyle factors, chronic diseases and medication, fat intake was positively associated with high AHI (≥20/h) (Q4 *vs.* Q1, RRR 2.98 (95% CI 1.20–7.38) (*p* for trend across quartiles was 0.046 across quartiles). Similarly, the association was not significant after further adjusting for total energy intake. BMI mediated 30% of the association between fat intake and AHI (direct effect 0.07, indirect effect 0.03, *p* < 0.05) (Table S2 and Figure S3). However, BMI did not mediate the association between fat intake and daytime sleepiness (Table S3). 

## 4. Discussion

To the best of our knowledge, this is the first study to assess the association between macronutrient intake and sleep in a large population based cross-sectional study using objectively measured polysomnography. We found that high intake of fat was associated with daytime sleepiness and high AHI. The associations between fat intake and AHI was mediated by BMI. 

Although the mechanism of the associations between macronutrient intake and sleep parameters is yet to be clear, some possibilities have been suggested by previously published work. Sleep can be regulated by various hormones that is induced by food intake through communications between hypothalamus and the brain [17]. Both dietary carbohydrates and protein can affect tryptophan metabolism through the availability tryptophan uptake into the brain via the blood brain barrier [18]. Regarding the mechanism of fat intake and sleep parameters, it is suggested that fat may affect sleep by altering circadian regulation of hormonal, central nervous and metabolic systems [19]. 

We found a positive association between high fat intake and daytime sleepiness. Early experimental studies showed that both infusion of lipid into the small intestine and isoenergetic meals may cause a decline in alertness and concentration [20]. Wells et.al have shown that healthy young subjects felt sleepier and less awake 2–3 h after a high-fat-low-carbohydrate meal [21]. Although carbohydrate rich meals have been demonstrated to be associated with postprandial lassitude [22], a greater decline was seen in high fat intake [20]. Other laboratory evidences suggested the potential role of gut neuro hormones in promoting hypnogenesis through vagal activation which essentially triggers fatigue [23,24,25,26,27]. However, we did not have data on the timing of fat intake, and dietary data collection was prior to sleep measurements, so the immediate effect of sleepiness of high-fat diet was not able to be assessed. Long-term high fat intake may lead to elevated levels of leptin and decreased levels of ghrelin [28], which could regulate arousal and wakefulness via orexin [29]. Increased sleepiness was observed in mice with high-diet fed induced obesity [30]. In large scale studies, positive associations between obesity and excessive daytime sleepiness has been reported [31,32]. This is consistent with our data that participants in the obese group had a higher risk of daytime sleepiness after adjusting for lifestyle factors (Table S4). However, obesity does not seem to be a mediator of the association between fat intake and daytime sleepiness (Table S3).High fat intake was also found to be associated with a high level of AHI (≥20/h) in this study, after adjusting for age, waist, lifestyle factors, chronic diseases and medication. Similarly, previous experimental studies found a fatty meal the night before bed would increase AHI in OSA patients [4]. Long-term effect of high-fat diet on AHI is not clear. In non-obese rats, high-fat fed diet increases apnoea, and this could be reversed and prevented by a low dose injection of metformin (a drug for insulin resistance) [33]. This may suggest that insulin resistance induced by high fat diet may be one of the mechanisms leading to increased AHI, but was dependent on body weight. In patients with type 2 diabetes, AHI (≥30/h) was associated with higher BMI [34]. Obesity has been suggested as one of the main risk factors of sleep apnoea [35] in the literature. In our study, being obese was strongly associated with higher AHI compared with non-obese participants (Table S5). Our mediation modelling suggests that the direct effect of BMI on AHI was about five times stronger than the effect from fat intake, and about 30% of the effect on AHI comes from BMI (Table S2 and Figure S3). 

Regarding energy intake, higher energy intake was associated with high level of AHI in our study (Table S5), and our sensitivity analysis suggested that it was a confounder in the association between fat intake and AHI and daytime sleepiness. However, energy intake estimated from self-reported dietary intake has been suggested to be less accurate [36]. Moreover, soft drink and alcohol were not included in the energy intake calculation in our study. 

The main merits of this study are: (1) it is the first investigation of the association between macronutrient intake and PSG measured sleep parameters as well as self-reported sleep problems in a relatively large sample; (2) we were able to adjust for a wide range of covariates including age, waist circumference, energy intake, education, smoking, alcohol intake, physical activity, shift work, depression, diabetes and medication. 

Several limitations in our study need to be acknowledged. Firstly, asynchronous exploration between macronutrient and sleep were performed due to the mismatch of time of the PSG study and dietary survey. Secondly, due to the nature of the cross-sectional study, causation cannot be made. Thirdly, because the study only involved men, the findings may not be generalised to women. In addition, we only conducted one overnight PSG assessment as it is not practical to have multiple night PSG assessments in large epidemiological studies. Despite objective sleep measurement, dietary intake was estimated by FFQ, rather than 24-h food recall or actual weighing. 24-h food recall provides meal specific food intake information, which has been suggested to be associated with circadian adaption [37]. However, it is impractical to conduct 24-h food recall in studies with large sample size, and 24-h recall does not capture a long term dietary habit as FFQ does.

In conclusion, high fat intake was associated with daytime sleepiness and high AHI. BMI mediates the association between fat and AHI but not daytime sleepiness. Although a public health benefit is suggested, future studies are needed to confirm the findings at the population level.

## Figures and Tables

**Table 1:** Characteristics of subjects according to quartiles of each macronutrient intake (*n* = 1815) ^1^.

Factors	Carbohydrate Intake (g)			Protein Intake (g)			Fat Intake (g)		*p*-Value
	Q1 (*n* = 454)	Q4 (*n* = 453)	*p*-Value	Q1 (*n* = 454)	Q4 (*n* = 453)	*p*-Value	Q1 (*n* = 454)	Q4 (*n* = 453)	
Age (years), mean (SD)	60.5 (11.7)	58.5 (11.4)	0.07	61.5 (12.1)	58.4 (10.9)	<0.001	59.9 (11.6)	59.5 (11.1)	0.47
Energy intake (kcal), mean (SD)	1539.1 (342.1)	2930.5 (606.7)	<0.001	1548.3 (348.4)	2900.5 (618.8)	<0.001	1535.1 (328.4)	2934.2 (596.9)	<0.001
Carbohydrates (g/day), mean (SD)	132.9 (26.0)	320.1 (91.5)	<0.001	157.5 (49.8)	283.1 (97.1)	<0.001	162.2 (51.2)	276.1 (93.0)	<0.001
Fat (g/day), mean (SD)	71.3 (22.2)	119.0 (34.7)	<0.001	66.5 (19.4)	123.4 (32.0)	<0.001	58.4 (10.9)	135.2 (25.8)	<0.001
Protein (g/day), mean (SD)	74.5 (23.1)	126.8 (37.1)	<0.001	64.1 (12.0)	141.6 (32.9)	<0.001	71.9 (19.0)	131.0 (39.0)	<0.001
Fibre (g/day), mean (SD)	18.4 (5.9)	37.7 (11.5)	<0.001	19.6 (7.3)	35.6 (11.0)	<0.001	21.2 (8.3)	34.3 (10.9)	<0.001
Body mass index (BMI), *n* (%)			0.71			0.003			0.49
<25	81 (18.7)	79 (18.2)		102 (23.4)	71 (16.3)		79 (18.2)	81 (18.6)	
25–30	214 (49.3)	207 (47.6)		211 (48.4)	201 (46.1)		214 (49.2)	192 (44.1)	
≥30	139 (32.0)	149 (34.3)		123 (28.2)	164 (37.6)		142 (32.6)	162 (37.2)	
Income, *n* (%)			0.08			<0.001			0.16
Low income	171 (39.1)	153 (34.2)		193 (44.3)	153 (34.3)		163 (37.1)	164 (36.5)	
Middle income	113 (25.9)	156 (34.9)		113 (25.9)	165 (37.0)		120 (27.3)	164 (36.5)	
High income	130 (29.7)	114 (25.5)		105 (24.1)	104 (23.3)		134 (30.5)	102 (22.7)	
Not stated	23 (5.3)	24 (5.4)		25 (5.7)	24 (5.4)		22 (5.0)	19 (4.2)	
Marriage status, *n* (%)			0.003			0.014			0.07
Married or living with a partner	323 (74.1)	342 (77.0)		316 (72.6)	343 (77.1)		351 (80.1)	324 (72.5)	
Separated/divorced	70 (16.1)	50 (11.3)		65 (14.9)	53 (11.9)		46 (10.5)	74 (16.6)	
Widowed	19 (4.4)	11 (2.5)		24 (5.5)	13 (2.9)		16 (3.7)	18 (4.0)	
Never married	22 (5.0)	40 (9.0)		28 (6.4)	33 (7.4)		24 (5.5)	30 (6.7)	
Not stated/refused	2 (0.5)	1 (0.2)		2 (0.5)	3 (0.7)		1 (0.2)	1 (0.2)	
Education, *n* (%)			0.07			0.18			0.10
≤High school	96 (25.3)	93 (23.3)		100 (27.0)	96 (24.2)		95 (25.1)	112 (28.1)	
Certificate	228 (60.2)	219 (54.9)		214 (57.8)	229 (57.8)		226 (59.6)	208 (52.1)	
Bachelor	52 (13.7)	83 (20.8)		50 (13.5)	69 (17.4)		53 (14.0)	75 (18.8)	
Not stated	3 (0.8)	4 (1.0)		6 (1.6)	2 (0.5)		5 (1.3)	4 (1.0)	
Current smoker, *n* (%)	71 (15.8)	51 (11.3)	0.22	62 (13.7)	66 (14.7)	0.35	48 (10.6)	61 (13.6)	0.36
Physical activity, *n* (%)			0.09			0.39			0.18
Sedentary	126 (30.6)	102 (24.2)		122 (29.4)	101 (24.0)		120 (28.7)	105 (24.9)	
Low exercise level	140 (34.0)	136 (32.2)		141 (34.0)	135 (32.1)		148 (35.4)	136 (32.3)	
Moderate exercise level	103 (25.0)	131 (31.0)		109 (26.3)	134 (31.8)		109 (26.1)	136 (32.3)	
High exercise level	43 (10.4)	53 (12.6)		43 (10.4)	51 (12.1)		41 (9.8)	44 (10.5)	
Depression, *n* (%)	37 (8.6)	56 (12.8)	0.17	33 (7.7)	61 (14.0)	0.016	38 (8.7)	64 (14.6)	0.029

^1^ Macronutrient intake was divided into quartiles. Q1 and Q4 stand for the lowest and highest quartile. The results presented are unadjusted.

**Table 2:** Polysomnography and self-reported sleep measures by quartiles of macronutrient intake in grams ^1^.

Sleep Parameters	Quartiles of Macronutrient Intake (g)				*p*-Value
	Carbohydrate Intake (g)				
**Polysomnography measures** (*n* = 784)	Q1 (*n* = 196)	Q2 (*n* = 196)	Q3 (*n* = 196)	Q4 (*n* = 196)	
Apnoea-Hypopnea Index (/h), *n* (%)					0.220
<5	48 (24.5)	40 (20.4)	49 (25.0)	32 (16.3)	
5–19	108 (55.1)	108 (55.1)	95 (48.5)	110 (56.1)	
≥20	40 (20.4)	48 (24.5)	52 (26.5)	54 (27.6)	
Total sleep duration (min), mean (SD)	376.8 (57.5)	376.7 (54.6)	369.1 (59.2)	369.7 (62.3)	0.380
**Self-reported measures**	Q1 (*n* = 372)	Q2 (*n* = 372)	Q3 (*n* = 372)	Q4 (*n* = 372)	
Daytime sleepiness (*n* = 1487), *n* (%)	133 (35.7)	160 (43.1)	159 (43.0)	152 (40.8)	0.320
Poor sleep quality (*n* = 773)^2^, *n* (%)	89 (48.4)	80 (42.6)	88 (46.1)	95 (50.5)	0.450
	Protein Intake (g)				
**Polysomnography measures** (*n* = 784)	Q1 (*n* = 196)	Q2 (*n* = 196)	Q3 (*n* = 196)	Q4 (*n* = 196)	
Apnoea-Hypopnea Index (/h), *n* (%)					0.230
<5	48 (24.5)	43 (21.9)	46 (23.5)	32 (16.3)	
5–19	104 (53.1)	109 (55.6)	105 (53.6)	103 (52.6)	
≥20	44 (22.4)	44 (22.4)	45 (23.0)	61 (31.1)	
TST (min), mean (SD)	374.6 (55.8)	375.8 (57.3)	365.4 (55.8)	376.5 (64.2)	0.200
**Self-reported measures**	Q1 (*n* = 372)	Q2 (*n* = 372)	Q3 (*n* = 372)	Q4 (*n* = 372)	
Daytime sleepiness (*n* = 1487), *n* (%)	131 (36.1)	164 (43.6)	152 (39.9)	157 (42.8)	0.490
Poor sleep quality (*n* = 773), *n* (%)	95 (51.4)	76 (40.0)	93 (49.7)	88 (46.6)	0.130
	Fat Intake (g)				
**Polysomnography measures** (*n* = 784)	Q1 (*n* = 196)	Q2 (*n* = 196)	Q3 (*n* = 196)	Q4 (*n* = 196)	
Apnoea-Hypopnea Index (/h), *n* (%)					0.004
<5	45 (23.0)	45 (23.0)	51 (26.0)	28 (14.3)	
5–19	117 (59.7)	100 (51.0)	101 (51.5)	103 (52.6)	
≥20	34 (17.3)	51 (26.0)	44 (22.4)	65 (33.2)	
TST (min), mean (SD)	374.4 (54.7)	373.2 (54.1)	375.8 (61.8)	368.8 (62.9)	0.660
**Self-reported measures**	Q1 (*n* = 372)	Q2 (*n* = 372)	Q3 (*n* = 372)	Q4 (*n* = 372)	
Daytime sleepiness (*n* = 1487), *n* (%)	137 (37.0)	151 (41.0)	144 (38.1)	172 (46.4)	0.051
Poor sleep quality (*n* = 773), *n* (%)	86 (45.5)	89 (46.8)	85 (46.4)	92 (48.7)	0.940

^1^ Data are presented by macronutrient intake in quartiles of grams (unadjusted). Q1–Q4 = quartiles of each macronutrient intake in grams. Macronutrient intake for polysomnography measurements presented are from those with polysomnography measurements (*n* = 784). Macronutrient intake for self-reported sleep parameters are from those with self-reported day time sleepiness data (*n* = 1487); ^2^ poor sleep quality was measured among those who had polysomnography measurements (*n* = 784), macronutrient intake refers to polysomnography measured.

**Table 3:** The prevalence ratio (95% CI) for self-reported sleep parameters across quartiles of macronutrient intakes ^1^.

Self-reported Sleep Symptoms	Quartiles of Macronutrient Intake (g)				*n*
	Q1 (*n* = 372) ref	Q2 (*n* = 372)	Q3 (*n* = 372)	Q4 (*n* = 372)	
**Daytime sleepiness ^2^**					
*Carbohydrate*					
Model 1	1.00	1.60 (1.08–2.37) *	1.69 (1.10–2.58) *	1.48 (0.89–2.46)	1487
Model 2	1.00	1.58 (1.02–2.46) *	1.40 (0.87–2.26)	1.33 (0.75–2.35)	1195
Model 3	1.00	1.46 (0.92–2.31)	1.25 (0.77–2.04)	1.19 (0.66–2.13)	1147
Model 4	1.00	1.31 (0.81–2.12)	1.05 (0.61–1.81)	0.85 (0.41–1.78)	1147
*Protein*					
Model 1	1.00	1.62 (1.09–2.40) *	1.29 (0.86–1.94)	1.59 (1.01–2.51) *	1487
Model 2	1.00	1.75 (1.13–2.74) *	1.32 (0.84–2.08)	1.74 (1.04–2.89) *	1195
Model 3	1.00	1.51 (0.96–2.40)	1.29 (0.81–2.06)	1.62 (0.96–2.74)	1147
Model 4	1.00	1.47 (0.91–2.36)	1.21 (0.71–2.05)	1.44 (0.73–2.86)	1147
*Fat*					
Model 1	1.00	1.53 (1.04–2.24) *	1.23 (0.83–1.80)	1.95 (1.28–2.99) **	1487
Model 2	1.00	1.59 (1.03–2.46) *	1.23 (0.80–1.87)	1.85 (1.15–2.96) *	1195
Model 3	1.00	1.53 (0.98–2.40)	1.12 (0.72–1.72)	1.78 (1.10–2.89) *	1147
Model 4	1.00	1.56 (0.97–2.53)	1.16 (0.69–1.95)	1.90 (0.93–3.91)	1147
**Poor sleep quality**					
*Carbohydrate*					
Model 1	1.00	0.89 (0.65–1.21)	0.97 (0.69–1.36)	1.08 (0.73–1.59)	751
Model 2	1.00	0.88 (0.61–1.27)	0.96 (0.66–1.40)	0.98 (0.62–1.54)	590
Model 3	1.00	0.90 (0.62–1.31)	0.94 (0.64–1.39)	0.95 (0.60–1.53)	569
Model 4	1.00	0.86 (0.58–1.28)	0.88 (0.57–1.36)	0.84 (0.47–1.51)	569
*Protein*					
Model 1	1.00	0.76 (0.56–1.04)	0.94 (0.69–1.28)	0.86 (0.60–1.23)	751
Model 2	1.00	0.77 (0.54–1.12)	0.92 (0.65–1.32)	0.89 (0.59–1.34)	590
Model 3	1.00	0.77 (0.53–1.13)	0.87 (0.60–1.26)	0.83 (0.55–1.27)	569
Model 4	1.00	0.74 (0.50–1.08)	0.79 (0.52–1.19)	0.69 (0.40–1.19)	569
*Fat*					
Model 1	1.00	1.03 (0.76–1.39)	1.02 (0.75–1.39)	1.07 (0.77–1.49)	751
Model 2	1.00	1.12 (0.79–1.60)	1.08 (0.76–1.55)	1.11 (0.75–1.63)	590
Model 3	1.00	1.06 (0.74–1.53)	0.98 (0.68–1.42)	1.01 (0.68–1.51)	569
Model 4	1.00	1.01 (0.69–1.48)	0.90 (0.59–1.38)	0.86 (0.49–1.51)	569

^1^ Poisson regression was performed for self-reported poor sleep quality and incidence rate ratio is presented; ^2^ multinomial logistic regression was performed for daytime sleepiness as it has three levels: “yes”, “sometimes”, and “no”, and the results were showing those who answered “yes” compared with “no”. Four models adjusted for different covariates are presented. Model 1: adjusted for age. Model 2: further adjusted for education (high school, certificate and bachelor), smoking (yes/no), alcohol intake (standard drinks 0, 1, 3), physical activity (sedentary, low, moderate and high), shift work (yes/no). Model 3: further adjusted for waist circumference (continuous), depression (yes/no), diabetes (yes/no), and medication (continuous). Model 4: further adjusted for energy intake. * *p* < 0.05, ** *p* < 0.01.

**Table 4:** The associations between macronutrient intake and Apnoea hypopnea index (AHI) ^1^.

AHI Categories	Models	Quartiles of Macronutrient Intake (g)				*n*
		Q1 (ref)	Q2	Q3	Q4	
**AHI (/h)**				***Carbohydrate***		
<5 (ref)	Model 1	1.00	1.00	1.00	1.00	169
5–19	Model 1	1.00	1.22 (0.72–2.06)	0.80 (0.45–1.41)	1.36 (0.67–2.74)	421
≥20	Model 1	1.00	1.36 (0.72–2.54)	0.96 (0.49–1.89)	1.27 (0.55–2.89)	194
						Subtotal: 784
<5 (ref)	Model 2	1.00	1.00	1.00	1.00	127
5–19	Model 2	1.00	1.79 (0.96–3.33)	1.21 (0.63–2.33)	1.77 (0.78–3.99)	338
≥20	Model 2	1.00	1.60 (0.76–3.38)	1.17 (0.54–2.54)	1.55 (0.60–4.02)	155
						Subtotal: 620
<5 (ref)	Model 3	1.00	1.00	1.00	1.00	123
5–19	Model 3	1.00	1.82 (0.94–3.52)	1.12 (0.57–2.21)	1.70 (0.73–3.95)	324
≥20	Model 3	1.00	1.44 (0.64–3.25)	1.07 (0.46–2.46)	1.47 (0.53–4.11)	149
						Subtotal: 596
<5 (ref)	Model 4	1.00	1.00	1.00	1.00	123
5–19	Model 4	1.00	1.59 (0.79–3.20)	0.87 (0.39–1.93)	1.15 (0.40–3.34)	324
≥20	Model 4	1.00	1.06 (0.45–2.49)	0.62 (0.24–1.60)	0.56 (0.16–2.05)	149
						Subtotal: 596
				***Protein***		
<5 (ref)	Model 1	1.00	1.00	1.00	1.00	169
5–19	Model 1	1.00	1.20 (0.72–2.01)	1.09 (0.64–1.85)	1.51 (0.79–2.87)	421
≥20	Model 1	1.00	1.09 (0.59–2.03)	1.04 (0.55–1.97)	1.80 (0.86–3.78)	194
						Subtotal: 784
<5 (ref)	Model 2	1.00	1.00	1.00	1.00	127
5–19	Model 2	1.00	1.44 (0.78–2.67)	1.18 (0.63–2.20)	1.96 (0.92–4.18)	338
≥20	Model 3	1.00	1.21 (0.57–2.54)	1.00 (0.48–2.12)	2.40 (1.00–5.76) *	155
						Subtotal: 620
<5 (ref)	Model 3	1.00	1.00	1.00	1.00	123
5–19	Model 3	1.00	1.22 (0.64–2.32)	0.99 (0.51–1.89)	1.63 (0.74–3.56)	324
≥20	Model 3	1.00	1.03 (0.46–2.32)	0.83 (0.36–1.87)	2.03 (0.79–5.22)	149
						Subtotal: 596
<5 (ref)	Model 4	1.00	1.00	1.00	1.00	123
5–19	Model 4	1.00	1.09 (0.55–2.14)	0.79 (0.37–1.69)	1.13 (0.41–3.10)	324
≥20	Model 4	1.00	0.83 (0.36–1.93)	0.54 (0.21–1.38)	0.99 (0.29–3.32)	149
						Subtotal: 596
				***Fat***		
<5 (ref)	Model 1	1.00	1.00	1.00	1.00	169
5–19	Model 1	1.00	0.85 (0.51–1.40)	0.74 (0.45–1.23)	1.25 (0.68–2.30)	421
≥20	Model 1	1.00	1.49 (0.80–2.77)	1.09 (0.58–2.06)	2.46 (1.21–5.00) *	194
						Subtotal: 784
<5 (ref)	Model 2	1.00	1.00	1.00	1.00	127
5–19	Model 2	1.00	0.84 (0.46–1.55)	0.67 (0.37–1.21)	1.33 (0.65–2.73)	338
≥20	Model 3	1.00	1.61 (0.77–3.40)	1.10 (0.52–2.30)	2.67 (1.15–6.20) *	155
						Subtotal: 620
<5 (ref)	Model 3	1.00	1.00	1.00	1.00	123
5–19	Model 3	1.00	0.83 (0.44–1.55)	0.66 (0.36–1.22)	1.40 (0.66–2.96)	324
≥20	Model 3	1.00	1.54 (0.69–3.46)	1.20 (0.54–2.67)	2.98 (1.20–7.38) *	149
						Subtotal: 596
<5 (ref)	Model 4	1.00	1.00	1.00	1.00	127
5–19	Model 4	1.00	0.67 (0.34–1.33)	0.46 (0.21–1.00) *	0.76 (0.26–2.23)	334
≥20	Model 4	1.00	1.25 (0.53–2.97)	0.84 (0.32–2.21)	1.63 (0.45–5.90)	154
						Subtotal:596

^1^ The results were from multinomial logistic regression. It presents comparing with the lowest level of sleep outcome, the relative risk ratio for medium or high level of having higher quartile of each macronutrient intake comparing with the lowest quartile of intake (Q2–4 *vs.* Q1). Four models adjusted for different covariates are presented. Model 1: adjust for age. Model 2: further adjusted for education (high school, certificate and bachelor), smoking (yes/no), alcohol intake (standard drinks 0, 1, 3), physical activity (sedentary, low, moderate and high), shift work (yes/no). Model 3: further adjusted for waist circumference (continuous), depression (yes/no), diabetes (yes/no), and medication (continuous). Model 4: further adjusted for energy intake. * *p* < 0.05.

## Supplementary Materials

The following are available online at http://www.mdpi.com/2072-6643/8/4/207s1.
