# Supplementary material for: Associations between Macronutrient Intake and Obstructive Sleep Apnoea as Well as Self-Reported Sleep Symptoms: Results from a Cohort of Community Dwelling Australian Men
Source: Nutrients. 2016 Apr 8;8(4):207. doi: 10.3390/nu8040207 (PMC4848676; doi:10.3390/nu8040207)
Supplement: Supplementary file 1 [file nutrients-08-00207-s001.docx]

Supplementary Materials: Associations between Macronutrient Intake and Obstructive Sleep Apnoea as Well as Self-Reported Sleep Symptoms: Results from a Cohort of Community Dwelling Australian Men

Yingting Cao, Gary Wittert, Anne W. Taylor, Robert Adams and Zumin Shi


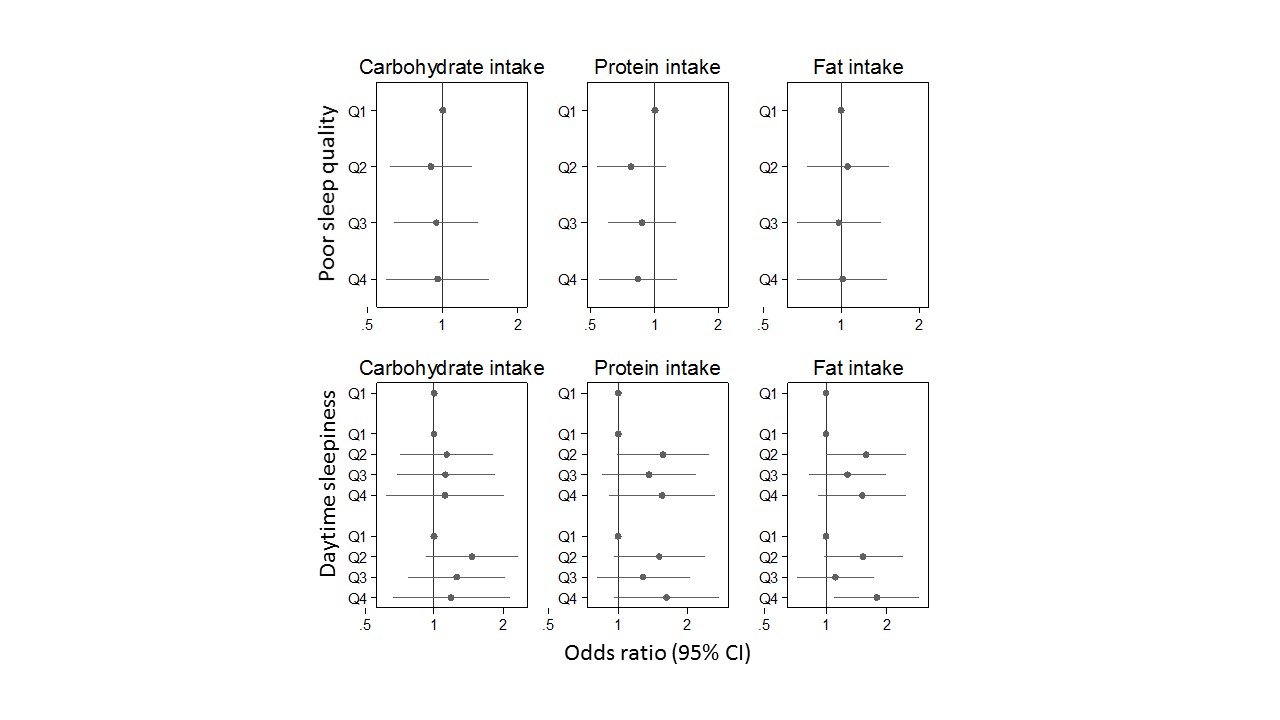


**Figure S1.** Associations between macronutrient intake and self-reported sleep parameters. Model 3 was used, adjusting for age, education (high school, certificate and bachelor), smoking (yes/no), alcohol intake (standard drinks 0, 1, 3), physical activity (sedentary, low, moderate and high), shift work (yes/no), waist circumference (continuous), depression (yes/no), diabetes (yes/no), and medication (continuous).


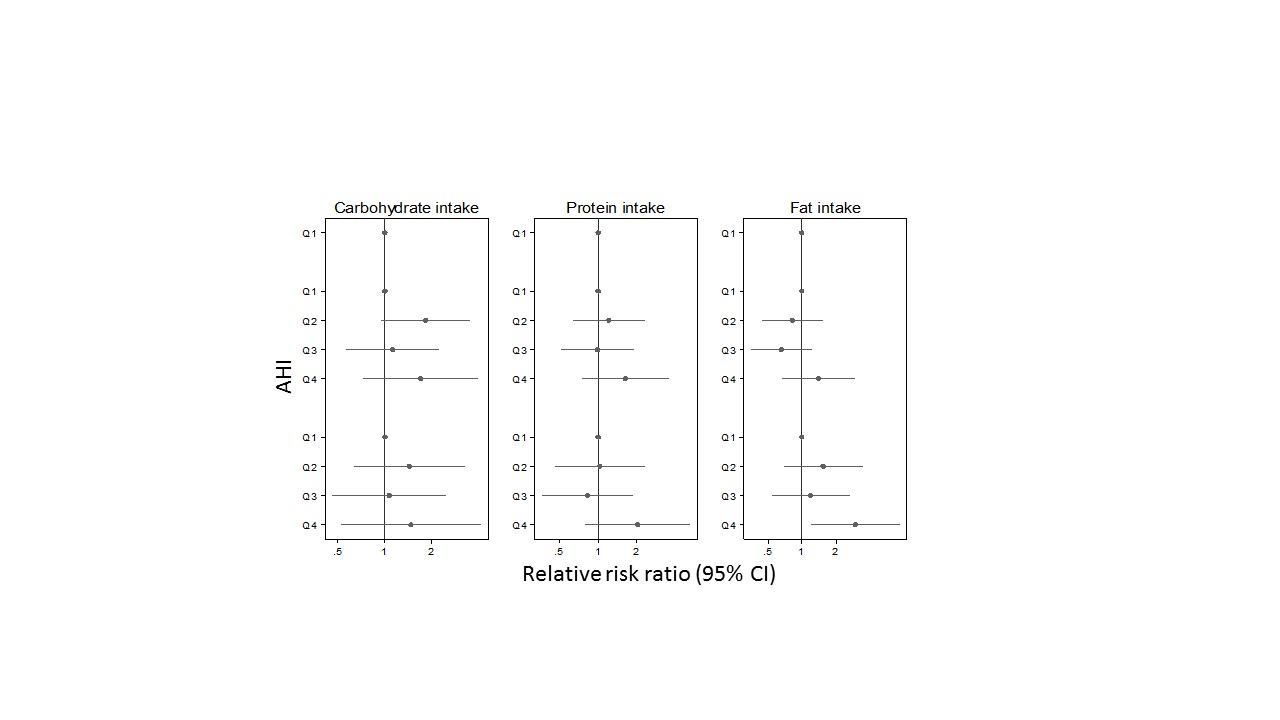


**Figure S2.** Associations between macronutrient intake and apnoea-hypopnea index (AHI).. Model 3 was used, adjusting for age, education (high school, certificate and bachelor), smoking (yes/no), alcohol intake (standard drinks 0, 1, 3), physical activity (sedentary, low, moderate and high), shift work (yes/no), waist circumference (continuous), depression (yes/no), diabetes (yes/no), and medication (continuous).


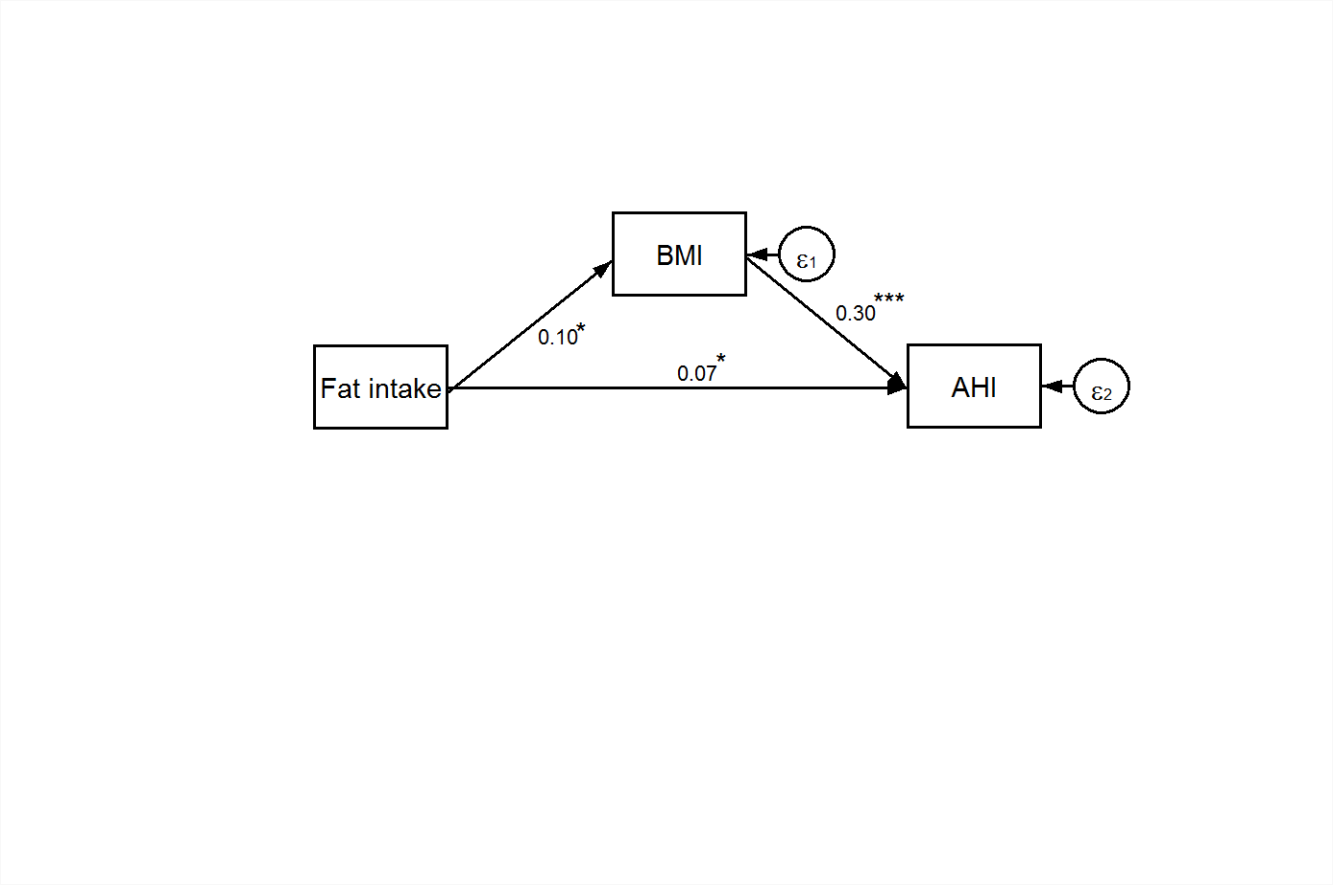


**Figure S3.** Standardized estimates for fat intake and body mass index (BMI). Fat intake was associated with BMI, which in turn was associated with AHI. The path coefficients, which can be interpreted as standardized beta weights in a regression model, are shown next to each arrow. All the coefficients shown were direct effects, indirect effects can be found in Table S1. Models were adjusted for age, education, smoking, alcohol consumption, physical activity and shift work. * *p* < 0.05; *** *p* < 0.001.

**Table S1.** Characteristics of PSG participants according to quartiles of each macronutrient intake (*n* = 784) ^1^**.**

| **Factors** | **Carbohydrate Intake (g)** | |  | **Protein Intake (g)** | |  | **Fat intake (g)** | | ***p*-Value** |
| --- | --- | --- | --- | --- | --- | --- | --- | --- | --- |
|  | **Q1(*n* = 196)** | **Q4 (*n* = 196)** | ***p*-Value** | **Q1 (*n* = 196)** | **Q4 (*n* = 453)** | ***p*-Value** | **Q1 (*n* = 196)** | **Q4 (*n* = 196)** |  |
| Age (years), mean (SD) | 59.5 (11.4) | 57.8 (10.3) | 0.34 | 60.3 (11.8) | 57.2 (10.2) | 0.017 | 59.1 (11.7) | 59.0 (10.7) | 0.29 |
| Energy intake (kcal), mean (SD) | 1515.2 (342.5) | 2913.2 (584.8) | <0.001 | 1526.4 (354.6) | 2926.7 (579.3) | <0.001 | 1522.2 (334.3) | 2933.3 (565.2) | <0.001 |
| Carbohydrates (g/day), mean (SD) | 132.9 (26.4) | 317.9 (82.6) | <0.001 | 155.7 (51.4) | 285.8 (94.6) | <0.001 | 162.5 (53.2) | 276.0 (87.6) | <0.001 |
| Fat (g/day), mean (SD) | 70.3 (22.2) | 118.5 (34.1) | <0.001 | 66.0 (19.3) | 125.6 (30.7) | <0.001 | 58.0 (11.1) | 134.9 (23.9) | <0.001 |
| Protein (g/day), mean (SD) | 72.0 (24.0) | 124.1 (32.8) | <0.001 | 62.2 (12.0) | 140.0 (29.5) | <0.001 | 69.3 (17.6) | 131.1 (35.8) | <0.001 |
| Fibre (g/day), mean (SD) | 18.0 (6.0) | 37.7 (11.0) | <0.001 | 19.4 (7.4) | 36.3 (10.9) | <0.001 | 21.1 (8.5) | 34.6 (10.8) | <0.001 |
| BMI, *n* (%) |  |  | 0.58 |  |  | 0.54 |  |  | 0.034 |
| <25 | 42 (21.8%) | 36 (18.6%) |  | 47 (24.2%) | 34 (17.5%) |  | 39 (20.1%) | 37 (19.2%) |  |
| 25–30 | 89 (46.1%) | 89 (45.9%) |  | 92 (47.4%) | 88 (45.4%) |  | 101 (52.1%) | 80 (41.5%) |  |
| ≥30 | 62 (32.1%) | 69 (35.6%) |  | 55 (28.4%) | 72 (37.1%) |  | 54 (27.8%) | 76 (39.4%) |  |
| Income, *n* (%) |  |  | 0.81 |  |  | 0.002 |  |  | 0.11 |
| Low income | 60 (31.4%) | 59 (30.1%) |  | 76 (39.8%) | 60 (30.6%) |  | 64 (33.3%) | 68 (34.7%) |  |
| Middle income | 58 (30.4%) | 66 (33.7%) |  | 56 (29.3%) | 74 (37.8%) |  | 55 (28.6%) | 75 (38.3%) |  |
| High income | 62 (32.5%) | 62 (31.6%) |  | 47 (24.6%) | 51 (26.0%) |  | 64 (33.3%) | 45 (23.0%) |  |
| Not stated | 11 (5.8%) | 9 (4.6%) |  | 12 (6.3%) | 11 (5.6%) |  | 9 (4.7%) | 8 (4.1%) |  |
| Marriage status, *n* (%) |  |  | 0.42 |  |  | 0.016 |  |  | 0.52 |
| Married or living with a partner | 148 (77.9%) | 151 (77.8%) |  | 139 (72.8%) | 155 (79.5%) |  | 158 (82.7%) | 146 (74.9%) |  |
| Separated/divorced | 21 (11.1%) | 19 (9.8%) |  | 25 (13.1%) | 20 (10.3%) |  | 15 (7.9%) | 30 (15.4%) |  |
| Widowed | 9 (4.7%) | 7 (3.6%) |  | 10 (5.2%) | 5 (2.6%) |  | 7 (3.7%) | 10 (5.1%) |  |
| Never married | 10 (5.3%) | 16 (8.2%) |  | 15 (7.9%) | 14 (7.2%) |  | 10 (5.2%) | 9 (4.6%) |  |
| Not stated/refused | 2 (1.1%) | 1 (0.5%) |  | 2 (1.0%) | 1 (0.5%) |  | 1 (0.5%) | 0 (0.0%) |  |
| Education, *n* (%) |  |  | 0.23 |  |  | 0.25 |  |  | 0.28 |
| ≤High school | 29 (18.0%) | 37 (21.3%) |  | 36 (23.1%) | 39 (23.1%) |  | 33 (20.6%) | 41 (24.3%) |  |
| Certificate | 109 (67.7%) | 94 (54.0%) |  | 103 (66.0%) | 96 (56.8%) |  | 109 (68.1%) | 91 (53.8%) |  |
| Bachelor | 22 (13.7%) | 42 (24.1%) |  | 15 (9.6%) | 33 (19.5%) |  | 17 (10.6%) | 35 (20.7%) |  |
| Not stated | 1 (0.6%) | 1 (0.6%) |  | 2 (1.3%) | 1 (0.6%) |  | 1 (0.6%) | 2 (1.2%) |  |
| Current smoker, *n* (%) | 34 (17.4%) | 21 (10.8%) | 0.30 | 30 (15.4%) | 28 (14.4%) | 0.94 | 24 (12.3%) | 31 (15.9%) | 0.77 |
| Physical activity, *n* (%) |  |  | 0.35 |  |  | 0.16 |  |  | 0.88 |
| Sedentary | 57 (31.7%) | 49 (26.2%) |  | 57 (31.0%) | 40 (21.4%) |  | 57 (31.1%) | 43 (23.1%) |  |
| Low exercise level | 60 (33.3%) | 57 (30.5%) |  | 57 (31.0%) | 55 (29.4%) |  | 59 (32.2%) | 63 (33.9%) |  |
| Moderate exercise level | 45 (25.0%) | 56 (29.9%) |  | 49 (26.6%) | 67 (35.8%) |  | 47 (25.7%) | 60 (32.3%) |  |
| High exercise level | 18 (10.0%) | 25 (13.4%) |  | 21 (11.4%) | 25 (13.4%) |  | 20 (10.9%) | 20 (10.8%) |  |
| Depression, *n* (%) | 11 (6.0%) | 24 (12.5%) | 0.20 | 11 (6.0%) | 23 (12.1%) | 0.23 | 9 (4.8%) | 21 (11.2%) | 0.09 |

^1^ Macronutrient intake was divided into quartiles. Q1 and Q4 stand for the lowest and highest quartile. The results presented are unadjusted.

**Table S2.** Standardized effects of fat intake and BMI for AHI**.**

| **Outcome** | **Direct Effect** | **Indirect Effect** | **Total Effect** |
| --- | --- | --- | --- |
| BMI |  |  |  |
| Fat intake→BMI | 0.10 * | - | 0.10 * |
| AHI |  |  |  |
| Fat intake→AHI | 0.07 * | 0.03 * | 0.10 ** |
| BMI→AHI | 0.30 *** | - | 0.30 *** |

* *p* < 0.05, ** *p* < 0.01, and *** *p* < 0.001; Models were adjusted for age, education, smoking, alcohol consumption, physical activity and shift work.

**Table S3.** Standardized effects of fat intake and BMI for daytime sleepiness ^1^.

| **Outcome** | **Direct Effect** | **Indirect Effect** | **Total Effect** |
| --- | --- | --- | --- |
| BMI |  |  |  |
| Fat intake→BMI | 0.05 | - | 0.05 |
| AHI |  |  |  |
| Fat intake→Daytime sleepiness | 0.02 | 0.00 | 0.02 |
| BMI→Daytime sleepiness | −0.03 | - | −0.03 |

^1^ Daytime sleepiness was used as continuous variable in the structural equation model and total effect estimation (the answer to daytime sleepiness “yes” was assigned with value 2, “no” was assigned with value 0, “sometimes” was assigned with value 1). Models were adjusted for age, education, smoking, alcohol consumption, physical activity and shift work.

**Table S4.** The association (relative risk ratio 95% CI) between BMI and daytime sleepiness ^1^**.**

| **Daytime Sleepiness (ref = No)** | **Models** | **BMI (kg/m^2^)** | | | ***n*** |
| --- | --- | --- | --- | --- | --- |
|  |  | **Normal Weight (ref)** | **Overweight** | **Obese** |  |
| Sometimes | Model 1 | 1.00 | 1.14 (0.79–1.65) | 1.03 (0.69–1.53) | 1459 |
|  | Model 2 | 1.00 | 1.26 (0.83–1.91) | 1.04 (0.66–1.63) | 1179 |
| Yes | Model 1 | 1.00 | 1.05 (0.73–1.52) | 1.53 (1.04–2.25) * | 1459 |
|  | Model 2 | 1.00 | 1.29 (0.85–1.98) | 1.71 (1.10–2.67) * | 1179 |

^1^ Multinomial logistic regression was performed. Cut-offs for overweight and obesity were BMI 25 and 30 (kg/m^2^) respectively. Model 1: adjusted for age. Model 2: further adjusted for education (high school, certificate and bachelor), smoking (yes/no), alcohol intake (standard drinks 0, 1, 3), physical activity (sedentary, low, moderate and high), shift work (yes/no). * *p* < 0.05.

**Table S5.** The associations (relative risk ratio) between body weight and energy intake and apnoea-hypopnea index (AHI) ^1^.

| **AHI Categories** | **Models** | **BMI (kg/m^2^)** | | | ***n*** |
| --- | --- | --- | --- | --- | --- |
| AHI (/h) (<5 as ref) |  | Normal Weight (ref) | Overweight | Obese |  |
| 5–19 | Model 1 | 1.00 | 1.23 (0.80–1.89) | 2.60 (1.53–4.44) ** | 775 |
|  | Model 2 | 1.00 | 1.45 (0.87–2.42) | 2.45 (1.32–4.54) ** | 615 |
| ≥20 | Model 1 | 1.00 | 2.03 (1.10–3.76) * | 9.63 (4.89–18.97) ** | 775 |
|  | Model 2 | 1.00 | 3.35 (1.52–7.37) ** | 14.24 (6.09–33.29) ** | 615 |
|  |  |  | **Energy intake (kcal)** |  |  |
| AHI (/h) <5 (ref) |  | Low (ref) | Medium | High |  |
| 5–19 | Model 1 | 1.00 | 0.66 (0.43–1.00) | 1.53 (0.94–2.47) | 784 |
|  | Model 2 | 1.00 | 0.60 (0.36–1.00) * | 1.17 (0.67–2.04) | 620 |
| ≥20 | Model 1 | 1.00 | 0.80 (0.48–1.34) | 2.56 (1.47–4.43) ** | 784 |
|  | Model 2 | 1.00 | 0.70 (0.38–1.30) | 2.14 (1.13–4.03) * | 620 |

^1^ Multinomial logistic regression was performed. Model 1: adjusted for age. Model 2: further adjusted for education (high school, certificate and bachelor), smoking (yes, no), alcohol intake (standard drinks 0, 1, 3), physical activity (sedentary, low, moderate and high), shift work (yes, no). * *p* < 0.05; ** *p* < 0.01.
